# Decompressive Hemicraniectomy in a South American Population – Morbidity and Outcomes Analysis

**DOI:** 10.1371/journal.pone.0146747

**Published:** 2016-01-14

**Authors:** Roberto Bezerra Vital, Pedro Tadao Hamamoto Filho, Gustavo Jose Luvizutto, Luis Gustavo Ducati, Gabriel Pereira Braga, Helio Rubens de Carvalho Nunes, Flavio Ramalho Romero, Eliana Marisa Ganem, Marco Antonio Zanini, Rodrigo Bazan

**Affiliations:** 1 Department of Neurology, Psychology and Psychiatry at Botucatu Medical School, Univ Estadual Paulista, Botucatu, SP, Brazil; 2 Department of Public Health Medicine at Botucatu Medical School, Univ Estadual Paulista, Botucatu, SP, Brazil; 3 Department of Anesthesiology at Botucatu Medical School, Univ Estadual Paulista, Botucatu, SP, Brazil; Fraunhofer Research Institution of Marine Biotechnology, GERMANY

## Abstract

**Background:**

Malignant cerebral artery strokes have a poor prognosis, with nearly 80% of mortality in some series despite intensive care. After a large randomized trial, decompressive hemicraniectomy has been performed more often in stroke patients. Here, we describe patients in a tertiary teaching hospital in Brazil, emphasizing the impact of age on outcomes.

**Methods:**

A retrospective cohort of patients, with malignant strokes which received a decompressive hemicraniectomy, from paper and electronic medical records, from January 2010 to December 2013 was divided into two groups according to age.

**Results:**

The final analysis included 60 patients. The overall mortality was higher among patients older than 60 yrs (67% vs. 41%; p = 0.039), whose group also had a worse outcome (76% with mRS 5 or 6) at 90 days (OR 3.91 CI95% 1.30–11.74), whereas only 24% had mRS of 0–4 (p = 0.015). All patients who presented with sepsis died (p = 0.003). The incidence of pulmonary infection was very high in the elderly group (76%) with significant intergroup differences (p = 0.027, OR 8.32 CI95% 0.70–98.48).

**Conclusions:**

Older patients present more commonly with infections, more disabilities and a higher mortality, highlighting very poor results in elderly population. These results should be proved with a South American trial, and if confirmed, it can impact on future decisions regarding decompressive craniectomy for acute ischemic stroke in our region.

## Introduction

Ischemic strokes account for 87% of all strokes[1], and occlusion of the middle cerebral artery (MCA) can lead to malignant infarctions. Cerebral edema quickly develops, leading to life-threatening situations[2]. MCA infarctions have a poor prognosis, with a mortality rate of nearly 80% in some series[3,4] despite intensive care.

Medical intervention with osmotic and anti-edema agents in these cases did not prove effective[5,6], leaving decompressive hemicraniectomy with duraplasty as the only effective treatment[7,8]. Three recent European trials, the HAMLET[9], DECIMAL[10] and DESTINY[11] trials, have demonstrated diminished mortality rates without increasing the proportion of severely disabled patients (mRS 5 or 6)[12]. All three controlled trials had an age limit of 60 years for inclusion. More recently the DESTINY II[13] trial investigated how hemicraniectomy affects patients older than 60 years of age.

Our aim was to analyze prognostic factors in patients with MCA malignant infarctions treated with decompressive hemicraniectomy in a Brazilian tertiary teaching hospital.

## Methods

### Patients

A retrospective cohort was assembled with data collected from paper and electronic medical records of patients who underwent a decompressive hemicraniectomy for MCA malignant infarctions between January 2010 and December 2013 in the Hospital of Clinics in Botucatu, São Paulo, Brazil, after approval by the ethics committee in human clinical research of the Botucatu Medical School (certificate supplied to editorial staff). All participants or they legal representatives agreed for this research by a written informed consent. The research authors kept all informed consents after the ethics committee approval.

Patients were divided into the following two age groups: < 60 years and ≥ 60 years. The baseline characteristics collected included age, gender, race, initial NIHSS (National Institutes of Health Stroke Scale) on admission, time from ictus to surgery, side affected, and comorbidities such as hypertension, diabetes, smoking and atrial fibrillation.

All patients underwent at least one initial CT scan. Surgery was performed according to a local protocol, as proposed by Vahedi *et al*.[12].

### Inclusion criteria

The inclusion criteria for surgery were as follows: clinical deficits suggestive of infarction in the MCA territory; decrease in the level of consciousness; definite or early signs on CT of an infarct of at least 2/3 of the MCA territory, with or without additional infarction in the territory of the anterior or posterior cerebral artery on the same side.

### Exclusion criteria

The exclusion criteria were as follows: prior mRS ≥2; signs of irreversible brainstem dysfunction (two fixed dilated pupils); contralateral ischemia; large hemorrhagic transformation of the infarct (≥parenchymal hemorrhage grade 2); short life expectancy; other serious illnesses that could affect outcome; known coagulopathy or systemic bleeding disorder; and high risk clinical conditions for anesthesia. Patients with a mydriatic pupil were assessed individually and were not excluded for surgical treatment consideration (at out protocol) or final statistical analysis.

None of the thrombolyzed patients were excluded in our series. Patients with concomitant ACA (anterior cerebral artery) or PCA (posterior cerebral artery) infarctions were excluded from the final analysis. Other exclusion criteria used included patients ≤ 18 years old, patients lost to follow-up and patients with incomplete data.

### Outcomes

The primary outcomes were the modified Rankin Scale (mRS) dichotomized as 0–4 and 5–6 at 90 days. The secondary outcomes measured included hospitalization time, sepsis, pneumonia, urinary tract infection, mRS 0–3 and 4–6, death and symptomatic hemorrhagic transformation (hemorrhage as the predominant cause of a neurologic deterioration). All outcome data were collected after a clinical examination at an ambulatory care center.

### Interventions

Decompressive hemicraniectomy was adapted from recommendations of Jüttler *et al*.[14]. In summary, we used a reversed question mark shaped skin incision based at the ear, made from the ipsilateral ear to the occiput with curvature to the midline. Temporal muscle isolation was performed according to the surgeon’s preference. Facial nerve protection was not performed. A minimum diameter of 12 cm was used for bone flap removal. Temporal bone removal was performed to expose the middle fossa. In all patients, the dura was opened in such a manner that the entirety of the craniotomy area would allow for brain expansion. No brain amputation was necessary. None of the surgeries included internal decompression (dissection of necrotic/ischemic tissue) or implantation of ICP (intracranial pressure) probes. Duraplasty was performed in all cases using either autologous pericranium or Neuro-Patch^®^ (Aesculap—B. Braun, Tuttlingen, Germany). Subgaleal drains were placed to prevent postoperative hematomas and removed within 24 hours. All patients were transferred to the neurological ICU receiving antiedema therapy (except for hypothermia) in addition to control of BP, blood glucose and hemoglobin. Cranioplasty was scheduled after at least six weeks.

All patients received the same medical treatment for acute stroke and the post-operative care on the neurological ICU, with the same clinical protocols.

### Statistical analysis

Group comparison was made by simple logistic regression analysis for a better mRS outcome at 90 days, considering prior mRS and thrombolytic therapy as confounding factors. Pearson's chi-square test was used to relate age and mortality rates. Univariate analysis was utilized for correlation of the patient’s baseline morbidities. P values of less than 0.05 were considered statistically significant.

## Results

### Patient Population

The patients’ characteristics are summarized in Table 1. Sixty patients underwent surgical decompression for MCA strokes during the study period, with 55% (n = 33) being over the age of 60 years. Overall, 52% were males (n = 31, p = 0.113) and 87% were Caucasian (n = 52, p = 0.143) without significant differences, as shown in Table 2.

**Table 1:** Demographic data of overall sample.

Variable	n	%
Age (yr) ^(1)^	63 (32–83)	
NIHSS at admission ^(1)^	19 (10–27)	
NIHSS prior to surgery ^(1)^	21 (14–27)	
Ictus (hr) ^(1)^	27 (6–56)	
Sex (male)	31	52
Race		
White	52	87
Black	6	10
Asian	2	3
Prior stroke	9	15
Hypertension	40	67
Diabetes	17	28
Smoking	22	37
Atrial fibrillation	11	18
Stroke Side		
Right	34	57
Left	26	43
Time from Ictus to Surgery > 48hr	13	22
Thrombolysed Patients	11	18

^(1)^ Summary in median (minimum—maximum)

**Table 2:** Demographic characteristics, comorbidities, stroke side, clinical status at admission and treatment.

	< 60 years Group (n = 27)	≥ 60 years (n = 33)	
Characteristics	n (%)	n (%)	p Value
Age (yr)			
Median	47	68	
Range	32–57	61–83	
≥ 70 yr		15 (45)	
Sex			
Male	17 (63)	14 (42)	0.113
Female	10 (37)	19 (58)	
Race			
White	21 (78)	31 (94)	0.143
Black	4 (15)	2 (6)	
Asian	2 (7)	0 (0)	
Comorbidities			
Hypertension	14 (52)	26 (79)	0.028
Diabetes	3 (11)	14 (42)	0.010
Smoking	10 (37)	12(36)	0.957
Atrial Fibrillation	2 (7)	9 (27)	0.091
Stroke Side			
Right	15 (56)	19 (58)	0.875
Left	12 (44)	14 (42)	
NIHSS on admission			
Median	17.5	19.5	0.172
Range	10–25	12–27	
Time from Ictus to Surgery (hr)			
Median	34	30.5	0.618
Range	12–56	6–55	
Thrombolyzed Patients	2 (7)	9 (27)	0.091

The mean age of the younger patient group was 47 yrs (32–57) compared to 68 yrs in the older group (61–83). The most prevalent comorbidity in both groups was hypertension, which was slightly more prevalent among the older group (p = 0.028). Diabetes was also more prevalent in older patients (p = 0.010). The other comorbidities are detailed in Table 2. The stroke location did not differ statistically between the two groups (p = 0.875).

The mean NIHSS on admission was similar between the two groups with 17.5 (10–25) in the younger group and 19.5 (12–27) in the older group.

The time from ictus to surgery was also similar between the groups at 34 hr (12–56) and 30.5 hr (6–55) in the younger and older groups, respectively. A relatively high number of patients (total n = 11) were thrombolyzed before surgery: two in the younger group and 9 in the older group. Three of the eleven patients had partial recanalization, which was confirmed with DSA or CT angiography. Of these eleven patients who received thrombolytic therapy, three also received bridging therapy (IV + mechanical thrombectomy with different devices).

### Primary Outcomes

The outcomes of the overall samples and comparison groups are summarized in Tables 3 and 4. Primary outcome of mRS 0–4 at 90 days was observed in 56% of the patients aged 60 yrs or less and only in 24% of patients in the older (> 60 yrs) group. The difference was statistically significant (p = 0.015).

**Table 3:** Primary and secondary outcomes of the sample.

Variable	n	%
*Primary Outcomes*		
Modified Rankin Scale		
0–4	23	38
5–6	37	62
Death	33	55
*Secondary Outcomes*		
Modified Rankin Scale		
0–3	5	8
4–6	55	92
Sepsis	9	15
Pneumonia	38	63
Urinary Tract Infection	7	12
Hospitalization days ^(1)^	17 (1–101)	
Symptomatic Hemorrhagic Transformation	12	20

^(1)^ Summary in median (minimum—maximum)

**Table 4:** Primary and secondary outcomes of < 60 years and ≥ 60 years patients after decompressive hemicraniectomy.

	< 60 years (n = 27)	≥ 60 years (n = 33)	
Outcomes	n (%)	n (%)	p Value
Infections during hospitalization			
Sepsis	1 (4)	8 (24)	0.033
Pneumonia	13 (48)	25 (76)	0.027
Urinary Tract Infection	3 (11)	4 (12)	1.000
Hospitalization days			
Median	24.5	51.5	0.634
Range	1–48	2–101	
Symptomatic Hemorrhagic Transformation	6 (22)	6 (18)	0.948
Modified Rankin Scale			
0–2	2 (7)	0 (0)	0.039
3	3 (11)	0 (0)	
4	10 (37)	8 (24)	
5	1 (4)	3 (9)	
6 (death)	11 (41)	22 (67)	
0–4	15 (56)	8 (24)	0.015
5–6	12 (44)	25 (76)	
0–3	5 (19)	0 (0)	0.014
4–6	22 (81)	33 (100)	

**Fig 1:**
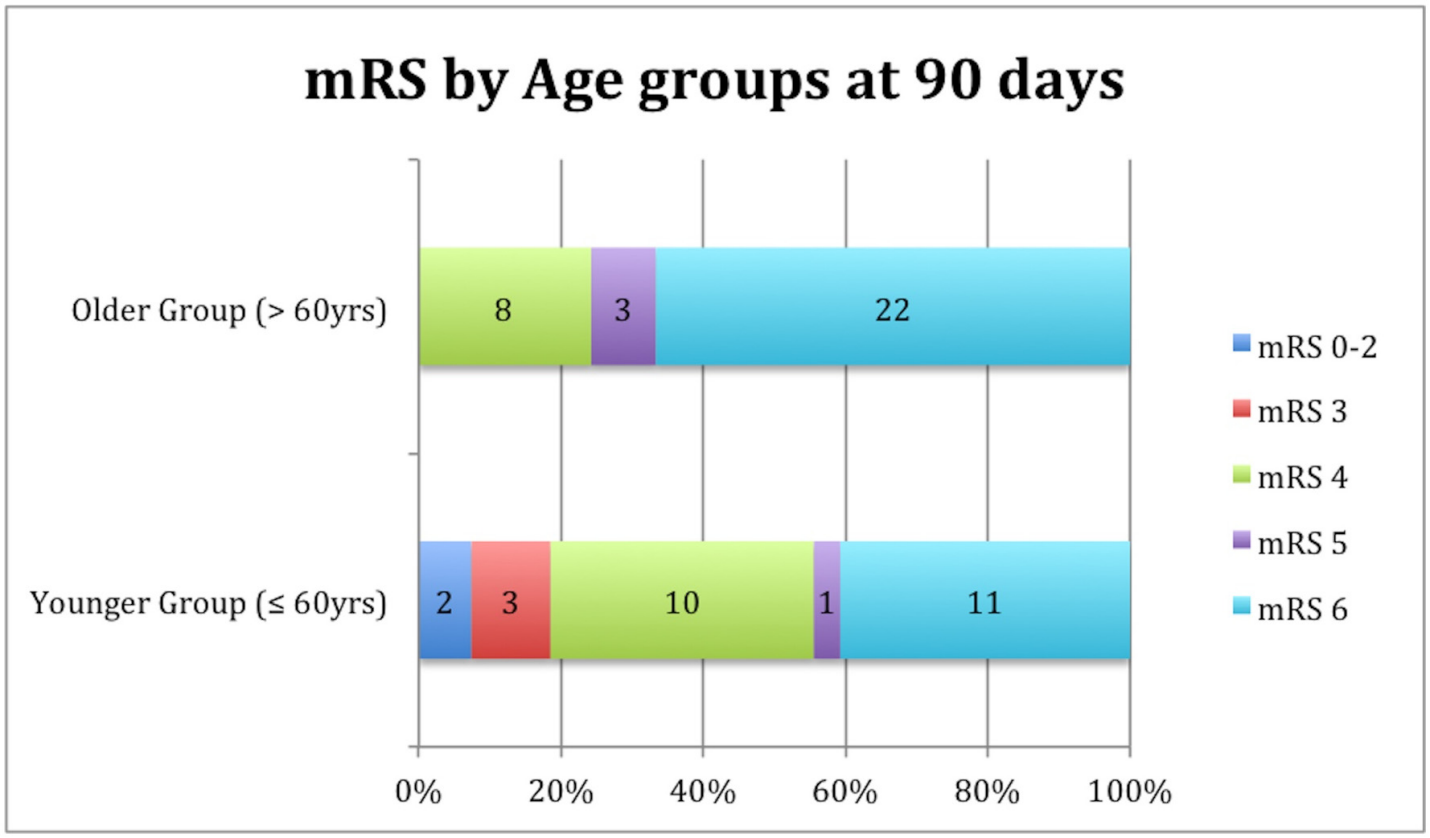
Functional outcome assessed by the modified Rankin score (mRS) at 90 days according to age group.

The mRS 5–6 outcome of 44% in the younger group also differed significantly (p = 0.015, OR 3.91 CI95% 1.30–11.74) from the 76% observed among the older patients, as displayed in Table 4. Overall, none of the patients had an mRS of 0 or 1. The mortality rates were statistically higher (Fig 1) in the >60 yrs group (p = 0.039).

### Secondary Outcomes

Among the 60 total patients, pneumonia was more prevalent in the older group (76%), and the difference was statistically significant (p = 0.027, OR 8.32 CI95% 0.70–98.48). Sepsis was also more prevalent in the older group, and the difference was also statistically significant (24%, p = 0.033). Considering both groups, all patients with sepsis died (p = 0.003).

The hospitalization times were longer in the ≥ 60 yrs group, averaging 51.5 days (2–101), but the difference was not statistically significant (p = 0.634). Symptomatic hemorrhagic transformation was similar in both groups (p = 0.948).

Analyzing the subgroups of mRS 0–3 and 4–6, the younger group had a better outcome, with 5 and 22 patients, respectively, whereas the older age group had no patients below mRS of 4 and 33 with mRS of 4–6.

We found no significant differences regarding the worse outcomes (mRS 5–6) between patients treated with thrombolysis and patients not treated with this therapy (p = 0.178).

There was no statistically significant difference comparing the time from initial stroke symptoms to surgical decompression between 24 hr and 48 hr, for the analyzed outcomes (mRS 0–4 p = 0.749; mRS 4–6 p = 1.00; mRS 6 p = 0.925). The NIHSS on admission did not relate with the primary outcomes; however, the NIHSS prior to surgery was significantly associated with the mRS 5–6 (p<0.001, OR 1.53 CI95% 1.23–1.91) and death (p<0.001, OR 1.48 CI95% 1.21–1.82)

## Discussion

Malignant MCA infarctions have posed a therapeutic challenge for decades. Many authors have tried to establish decompressive hemicraniectomy as a standard treatment for this severe condition. Schwab *et al*.[15] concluded that outcomes of patients treated with craniectomy in severe ischemic hemispheric infarction are surprisingly good and may be further improved by early decompressive surgery. The early and invasive treatment of malignant strokes by decompressive hemicraniectomy has become almost a rule in selected cases after the large prospective and randomized trials in Europe. There are undoubtedly differences in population characteristics that lead to distinct strategies in other parts of the world such as Latin America, specifically in its undeveloped areas where therapies are far from state-of-the-art. Nevertheless, we are closing this gap.

Without exception, we found that all of the malignant stroke patients older than 60 yrs of age selected for our study had more comorbidities, a longer hospital stay, more infectious complications, and fared worse than the younger patients, and although not statistically significant all this differences are clinically and economically relevant deserving future attention.

Other patient characteristics did not differ notably between the two age groups, including sex, race, afflicted stroke side, NIHSS on admission or time from ictus to surgery (24 hr or 48 hr), which is consistent with the findings of other reports[16]. Nevertheless the NIHSS prior to surgery revealed a significant correlation with poor outcomes, OR 1.53 (p<0.001, CI95% 1.23–1.91) for mRS 5–6 and OR 1.48 (p<0.001, CI95% 1.21–1.82) for death.

It is important to highlight that 18.3% of patients underwent surgery after receiving r-TPA, including bridging therapy, whereas the Brazilian rates range from 1.1% to 14%[17,18]. This may be a result of increasing number of studies focusing on thrombolysis in the elderly population such as the IST3.[19].

Furthermore, we did not find any significant differences in outcome between patients treated with thrombolysis before surgery versus those who had not received thrombolytic therapy. Few research articles can be found regarding this issue. Schuss *et al*.[20] reported that decompressive hemicraniectomy in patients with intravenous thrombolysis is feasible, and Takeuchi *et al*.[21] concluded that decompressive hemicraniectomy may be safe and useful for space-occupying edema, even after IV r-tPA administration for acute stroke. It was not predictive of bleeding complications or outcome in patients undergoing the surgery.

There are no trials, cohorts or even case series reported from a Latin American population, comparing or at least demonstrating outcomes of decompressive hemicraniectomy in stroke patients. Our studied population had characteristics that were similar to those in the European trials[13]; however, the outcomes were different.

As for the 60 yr old and younger population in the present study, 56% had a mRS score of 0–4 at 90 days and 44% had a mRS score of 5–6. In comparison, Jüttler *et al*.[11] found 76% of patients of the same age that had undergone a hemicraniectomy had a mRS score of 0–4, whereas only 24% had a mRS score of 5 or 6.

The elderly population, defined in our analysis, as older than 60 yrs, only 24% having a mRS score of 0–4 at 90 days and 76% having a mRS score of 5–6, representing an OR of 3.91 (CI95% 1.30–11.74) for an mRS of 5 or 6. A recent trial, DESTINY II[13], focusing on this elderly population of stroke-afflicted patients, found better outcomes: mRS of 0–4 present in 38% in the hemicraniectomy group.

These comparisons may seem questionable. First and foremost, we analyzed our population only retrospectively. Second and no less important, our primary outcome was measured at 90 days during follow-up visits, whereas the main European trials[9–12] assessed the same outcomes at a 1-year follow-up. This longer follow-up may justify different outcomes found on these trials[4,16,22,23].

Some hypotheses can be inferred from the worse outcomes. The lower functional reserves from the elderly, and 45% of this group was above 70 years of age, resulted in a much higher infection rate, leading to longer hospitalization duration and excessive mortality rates. It is known that the Brazilian population in general lacks adequate resources for basic sanitary conditions and health promotion, which certainly has an impact on the final recovery for stroke patients.

The present work is one of the few known series reporting a South American population that underwent decompressive hemicraniectomy for malignant MCA strokes. Our results did not reproduce the major clinical trials outcomes, which had been measured with optimal conditions and population. The limitations of a retrospective cohort are mitigated by the representative sample size because there are still large discrepancies among similar studies. Only the groups that underwent the surgical procedures were compared with each other; thus, we cannot assert that decompressive hemicraniectomy lowers mortality as concluded in other studies[9–13].

It is important to note that the threshold age was 60 yrs, which was the same as in other reports; however, in our opinion, this cutoff age should be raised in the near future to 65 yrs or even 70 yrs as the world population is aging. Another particular feature is the multiethnic society we have in our country, which differs from the European population. Albeit, our multi-ethnic country, the majority of our population were considered as white (Caucasian) by the investigators with subjective criteria.

Unfortunately we could not measure post-surgery complications such as sinking skin flap syndrome or syndrome of the trephined as well as quality of life scales due to the lack of those records thereafter considering this as significant weakness of our work.

Higher disabilities are not accompanied by worse quality of life, as discussed in recent studies[24,25], future studies should focus more in emotional and psychological aspects of patients and caregivers than simplistic scales like mRS or NIHSS.

## Conclusions

In the context of a considerable gap in the resources between more developed countries and developing ones, we describe our experience with treating malignant MCA strokes with decompressive hemicraniectomy. Older patients present more commonly with infections, more disabilities and a higher mortality, highlighting very poor results in elderly population. These results should be proved with a South American trial, and if confirmed, it can impact on future decisions regarding decompressive craniectomy for acute ischemic stroke in our region.

## Supporting Information

S1 Table Supporting patient data information. (XLSX)
